# The association of spinal osteoarthritis with lumbar lordosis

**DOI:** 10.1186/1471-2474-11-1

**Published:** 2010-01-02

**Authors:** Michael Papadakis, Georgios Papadokostakis, Nikos Kampanis, Georgios Sapkas, Stamatios A Papadakis, Pavlos Katonis

**Affiliations:** 1 2nd Department of Orthopaedic Surgery, University of Athens, Athens, Greece; 2 Institute of Applied and Computational Mathematics, Foundation of Research and Technology Hellas (IACM-FORTH), Heraklion, Greece; 3 Georgios Papadokostakis, University Hospital of Heraklion, Heraklion, Greece; 4 1st department of Orthopaedic Surgery, University of Athens, Athens, Greece; 5 5th Department of Orthopaedic Surgery, "KAT" General Hospital, Athens, Greece; 6 Pavlos Katonis, Department of Orthopaedic Surgery, University of Crete, Heraklion, Greece

## Abstract

**Background:**

Careful review of published evidence has led to the postulate that the degree of lumbar lordosis may possibly influence the development and progression of spinal osteoarthritis, just as misalignment does in other joints. Spinal degeneration can ensue from the asymmetrical distribution of loads. The resultant lesions lead to a domino- like breakdown of the normal morphology, degenerative instability and deviation from the correct configuration. The aim of this study is to investigate whether a relationship exists between the sagittal alignment of the lumbar spine, as it is expressed by lordosis, and the presence of radiographic osteoarthritis.

**Methods:**

112 female subjects, aged 40-72 years, were examined in the Outpatients Department of the Orthopedics' Clinic, University Hospital of Heraklion, Crete. Lumbar radiographs were examined on two separate occasions, independently, by two of the authors for the presence of osteoarthritis. Lordosis was measured from the top of L_1 _to the bottom of L_5 _as well as from the top of L_1 _to the top of S_1_. Furthermore, the angle between the bottom of L_5 _to the top of S_1_was also measured.

**Results and discussion:**

49 women were diagnosed with radiographic osteoarthritis of the lumbar spine, while 63 women had no evidence of osteoarthritis and served as controls. The two groups were matched for age and body build, as it is expressed by BMI. No statistically significant differences were found in the lordotic angles between the two groups

**Conclusions:**

There is no difference in lordosis between those affected with lumbar spine osteoarthritis and those who are disease free. It appears that osteoarthritis is not associated with the degree of lumbar lordosis.

## Background

Spinal osteoarthritis is a common condition, affecting almost 80% of those aged 40 or above [1,2]. It has also been shown that radiographic osteoarthritis in any site is associated with decreased survival independent of age and other factors like diabetes, smoking, alcohol abuse, history of cardiovascular disease and hypertension [3].

Research so far has identified a number of risk factors that predispose to the occurrence of osteoarthritis. Of note is the impact of joint alignment on the development of degenerative changes. When the shape of a joint is abnormal, the stresses are unequally distributed on its parts [4]. This asymmetrical load distribution contributes to the development of more or less severe, focal or diffuse, degenerative changes [5].

**Figure 1:**
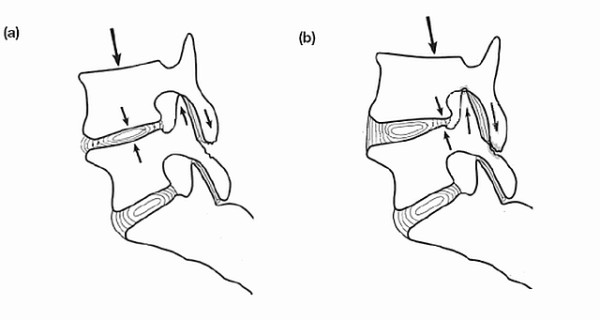
**The load distribution on the intervertebral disk and apophyseal joints is altered when the transmission of weight changes**.

The lumbar spine is a column, which is subjected to the compressive load exerted by the incumbent trunk. Its structure is ideally suited to withstand compressive loads [6,7]. The sagittal alignment influences the distribution of loads on spinal tissues [8-12]. Several investigators have argued that alterations in spinal balance and curvature are implicated in the development of early osteoarthritis and disc degeneration [11-17] (figure 1).

The development of degenerative changes adversely affects the normal morphology of the affected joints. In the lumbar spine, the changes observed are, amongst others, intervertebral space narrowing [18] especially in the anterior part [19], vertebral body osteophytosis and wedging, [17,20,21], loss of anterior column height [22] and hyperplastic modification of the facet joints [23,24]. Taken as a whole, these lesions lead to a domino- like breakdown of the normal morphology, degenerative instability and deviation from the correct configuration.

These published data indicate that the sagittal alignment of the lumbar spine influences the distribution of loads and accordingly, the development and progression of spinal osteoarthritis. The resultant lesions in turn induce a loss of stability and a progressive deformation of the proper configuration. The aim of the present study is to determine whether an association exists between osteoarthritis presence and the sagittal alignment of the lumbar spine, as it is expressed by lordosis. The hypothesis that is examined is that there is a significant difference between the mean magnitude of lumbar lordosis in patients with and without radiographic evidence of lumbar spine osteoarthritis.

## Methods

Participants in an ongoing epidemiological study of the prevalence of vertebral osteoporotic fractures formed the pool from which suitable subjects were selected. These participants are examined at the University Hospital of Heraklion, Crete. Part of their comprehensive evaluation is to have anteroposterior and lateral spine X-rays taken in the standing position, using the same procedure and equipment. The main reason for using the same subjects from the aforementioned study was to avoid exposing any further people to radiation. In addition, as those subjects were exclusively women of postmenopausal age, the average age of the subjects was in the period where the frequency of osteoarthritis becomes maximum [1,2]. Equally important, factors that are known to influence the sagittal curvature of the spine such as age and sex [25-28] would not confound the analysis.

All patients who had secondary osteoarthritis as well as patients whose lumbar curvature might have been altered from disease or iatrogenic intervention had to be excluded. Exclusion criteria were: 1) Congenital spinal diseases 2) Scoliosis 3) Spondylolisthesis - Spondylolysis 4) Vertebral fracture 5) History of spinal surgery 6) Inflammatory arthropathy 7) History of endocrine or metabolic disease.

All lumbar radiographs were examined on two separate occasions, independently, by two of the authors for the presence of features of osteoarthritis. The criteria used where those of Kellgren and Lawrence, and when evidence of two or more criteria were present, the diagnosis of lumbar osteoarthritis was made [29]. Interobserver agreement in detecting or excluding disease presence was 98%. If agreement was not reached, the patient was excluded from the study.

After the application of exclusion criteria, from 524 patients that were examined, only 145 were initially considered as potentially suitable. A further 33 patients were excluded after evaluation of spinal radiographs. The final sample consists of 112 postmenopausal women, aged 42-76 years old (mean 57.3 years).

After the designation of the final sample, lumbar lateral radiographs were digitized and measurements were made using the Cobb method with the assistance of a computer program. The use of computers for lumbar lordosis measurements has been shown to be at least equal, if not better, to the manual method [7,30,31]. Measurements were made from the top of L_1 _to the bottom of L_5 _as well as from the top of L_1 _to the top of S_1_. In addition, since several investigators have shown 50% to 75% of the total lordosis between L1 and S1 to be located at the bottom two motion segments [32-38], we also measured the angle between the bottom of L_5 _to the top of S_1_.

A priori power analysis showed that in order to have a power of 80% to detect a difference of as little as 10 degrees at the 0.05 level of significance assuming a standard deviation of 15 degrees, 35 women would be needed in each group. The increased enrolment improved the power of the study. Statistical analysis was performed using the one factor ANOVA model with no repeated measurements, chi - square test and for pairwise multiple comparisons, Ìann-Whitney test. All tests are two sided with p < 0.05 considered significant. The analysis was carried out using SPSS for Windows, Rel. 13.00. SPSS Inc. Chicago, IL.

The study protocol was approved by the Bioethics Board of the Faculty of Medicine, University of Crete. Written informed consent was obtained from all the subjects prior to their inclusion in the study.

## Results

**Figure 2:**
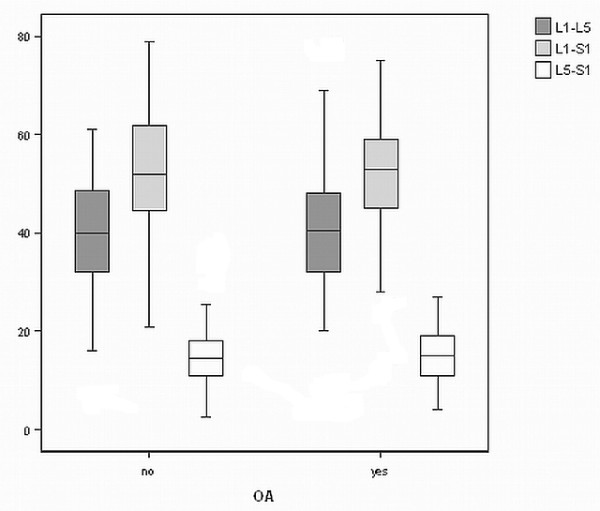
**Graph of the distribution of lordotic angles of the two groups**.

Forty- nine patients were diagnosed with radiographic osteoarthritis of the lumbar spine, while 63 patients had no evidence of the disease and served as controls. No statistically important differences were discovered in age (p= 0.309) and body build (p= 0.731), as it is expressed by body mass index (BMI). This demonstrates the homogeneity of the sample. Similarly, no statistically significant differences were found in lordosis angles between the groups. Additionally, the distribution of values was matched among the groups for all angles. Mean lordosis values for the entire cohort were L_1 _- L_5 _39.6^0 ^(95% confidence interval 42.05-37.23), L_1 _- S_1 _52.7^0 ^(95% confidence interval 55.16-50.28), L_5 _- S_1 _14.7^0 ^(95% confidence interval 15.8-13.56). These results are summarized in table 1 and figure 2 and are comparable with those reported in the literature [1,25-28]. To sum up, no relationship was found between the degree of lumbar lordosis and either the presence or absence of lumbar spine osteoarthritis.

**Table 1:** Age, BMI and lordotic angles of the total sample and the two groups.

	OA n = 49	NO OA n = 63	TOTAL SAMPLE n = 112	SIGNIFICANCE (p value)
**AGE (years)**	58.63 (59.97-57.29)	56.37 (57.63-55.11)	57.3 (58.61-55.99)	p = 0.309

**BMI (kg/m**^2^**)**	28.45 (29.35-27.55)	29.48 (30.28-28.68)	29.03 (29.88-28.18)	p = 0.732

**L1 - L5 (deg)**	39.53 (42.24-36.82)	39.73 (41.9-37.56)	39.64 (42.05-37.23)	p = 0.616

**L1 - S1 (deg)**	52.31 (54.69-49.93)	53.05 (55.55-50.55)	52.72 (55.16-50.28)	p = 0.672

**L5 - S1 (deg)**	14.54 (15.62-13.46)	14.80 (15.96-13.64)	14.68 (15.8-13.56)	p = 0.564

OA - Osteoarthritis of the lumbar spine. Numbers outside parentheses are means, numbers inside parentheses are 95% confidence intervals.

## Discussion

The clinical significance of the sagittal profile of the lumbar spine lies in its association with degeneration and low back pain. As already mentioned in the Introduction, several authors have argued that alterations in spinal balance and curvature are implicated in the development of disc degeneration and spinal osteoarthritis. This study was undertaken to elucidate the relationship, if any, between the sagittal curvature of the lumbar spine and the presence of osteoarthritis in the same area. Our results indicate that no such relationship exists.

When attempting to compare our results with those previously reported in the literature, a problem that comes up is the diversity of methods used to measure lordosis angles radiologically. Even when Cobb's method is used, different authors use different start and end points for measurements [30,38-41]. Another striking point is that the criteria as to what constitutes lumbar degenerative disease are often not expressly stated. This lack of standardization between reports causes difficulty in making exact comparisons.

The findings of the studies that have examined the correlation of lumbar osteoarthritis and lordosis are contradictory. Lin et al [41] measured lordosis in a sample of 149 symptom-free Chinese adults, 45 of which had some degree of osteoarthritis of their lumbar spine. They report no differences in lordosis between those with and without degenerative changes. Similarly, Lebkowski et al [42] did not find diminished lordosis in patients with lumbar degenerative disk disease. In contrast to these studies, where an association was not discovered, other investigators [38-40] report smaller lordosis and lumbosacral angles in patients with lumbar degenerative disease as compared to controls. Conversely, Farhni and Trueman [43] discovered smaller lordotic angles and lower incidence of degenerative changes in a cadaver sample of Indian men, compared with Caucasians. A number of studies have been conducted where radiographic evidence of lumbar osteoarthritis was present and lordosis was measured, but no attempt was made to investigate any relationship between the two [44-46].

The lack of statistically significant differences in our study can be partly explained by the fact that each person has a unique posture and spinal curvature. What constitutes deviation from the correct alignment and abnormal loading, that could induce degenerative changes on the lower spine, is probably a personalized characteristic. In a similar rationale, lumbar lordosis has a wide range of normal values, and any changes that might occur sooner or later may still be within this normal range. A limitation of the present study is that a cross - sectional rather than a prospective design was applied. Any future research on this subject should also examine the progression of disease of particular patients and the alteration of their individual spinal curves over time.

## Conclusions

**Figure 3:**
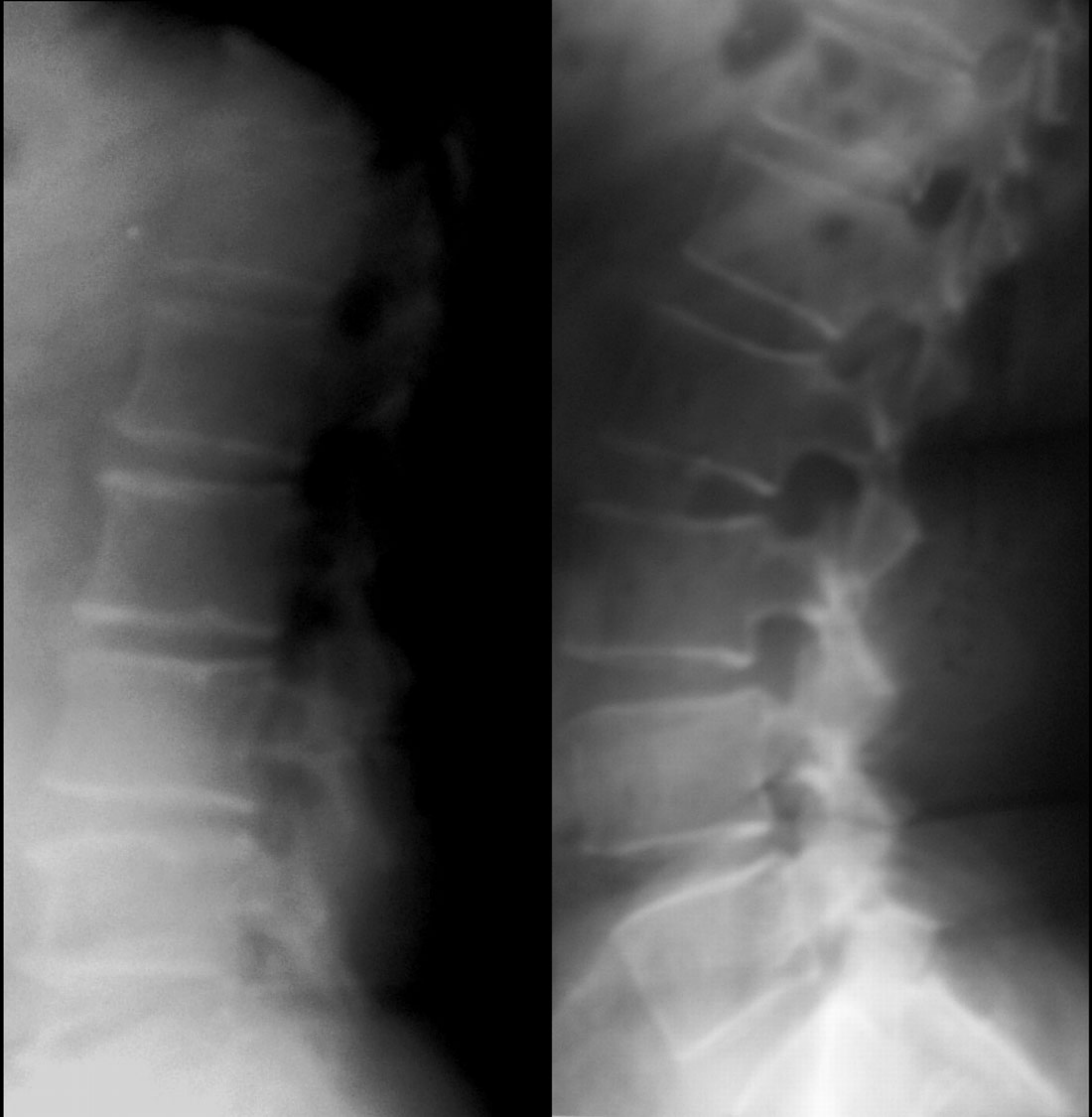
**Left: OA patient with minimal lordosis; L_1 _- L_5 _6^0^, L_1 _- S_1 _28^0^, L_5 _- S_1 _21^0^**. Right: OA patient with exaggerated lordosis; L _1_**- L**_5_**51**^0^**L**_1_**- S**_1_**70**^0^**L**_5_**- S**_1_**19**^0^.

In conclusion, no differences were found in lordosis between patients affected with lumbar spine osteoarthritis and those who are disease free. It appears that radiographic osteoarthritis is not associated with the degree of lumbar lordosis (figure 3). It is therefore suggested that lumbar lordosis is neither an outcome nor a contributing factor of spinal osteoarthritis.

## Competing interests

The authors declare that they have no competing interests.

## Authors' contributions

All authors participated in the conception and design of the study. MP and GP collected the data. All authors carried out data analysis and participated in the drafting of the manuscript.

## Pre-publication history

The pre-publication history for this paper can be accessed here:

http://www.biomedcentral.com/1471-2474/11/1/prepub
